# Larval development to the first eighth zoeal stages in the deep-sea caridean shrimp *Plesionika grandis* Doflein, 1902 (Crustacea, Decapoda, Pandalidae)

**DOI:** 10.3897/zookeys.719.20916

**Published:** 2017-12-07

**Authors:** Guo-Chen Jiang, Tin-Yam Chan, Tung-Wei Shih

**Affiliations:** 1 National Museum of Marine Science & Technology, Keelung 20248, Taiwan, R.O.C.; 2 Institute of Marine Biology and Center of Excellence for the Oceans, National Taiwan Ocean University, Keelung 20224, Taiwan, R.O.C.

**Keywords:** Deep-sea, larval development, Pandalidae, *Plesionika*, shrimps, zoea

## Abstract

The larvae of the deep-sea pandalid shrimp *Plesionika grandis* Doflein, 1902 were successfully reared in the laboratory for the first time. The larvae reached the eighth zoeal stage in 36 days, both of which are longest records for the genus. Early larval stages of *P. grandis* bear the general characters of pandalid shrimps and differ from the other two species of *Plesionika* with larval morphology known in the number of spines on the anteroventral margin of carapace, number of tubercles on antennule, endopod segmentation in antenna, and third maxilliped setation. Although members in *Plesionika* are often separated into species groups, members of the same species group do not necessarily have similar early larval morphology. Since the zoea VIII of *P. grandis* still lacks pleopods and fifth pereiopod, this shrimp likely has at least 12 zoeal stages and a larval development of 120 days.

## Introduction

The predominant deep-sea shrimp genus *Plesionika* Bate, 1888 is the most diverse genus in the caridean family Pandalidae Haworth, 1825, being represented by 93 species (Cardoso 2011; De Grave and Fransen 2011; Li and Chan 2013; Komai and Tsuchida 2014). Some of them are commercially important such as *P. izumiae* Omori, 1971, *P. martia* (A. Milne-Edwards, 1883), *P. narval* (Fabricius, 1787), and *P. quasigrandis* Chace, 1985 (Hayashi and Koike 1976; Holthuis 1980; Chilari et al. 2005; Chakraborty et al. 2015). Nevertheless, larval development in these shrimps has only been known in two species, namely *P. edwardsii* (Brandt, 1851) [zoea (hereafter with the abbreviation Z) I–VII; Landeira et al. 2009a] and *P. narval* [ZI–V, decapodid; Landeira et al. 2009b, Landeira et al. 2014], since rearing of deep-sea shrimps and their larvae are generally very difficult (Landeira et al. 2014).

*Plesionika grandis* Doflein, 1902 is a widely distributed species in the Indo-West Pacific from Japan to NE Australia and Madagascar at depths of 110–375 m (Chan and Crosnier 1991) and is rather common amongst the deep-sea catches in Taiwan. The present work succeeded in obtaining a live ovigerous female of *P. grandis* and maintained it in the laboratory until its eggs were hatched. Larvae developed into the eighth zoeal stage in 36 days, enabling the larval morphology of *P. grandis* is described and illustrated for the first time.

## Materials and methods

The ovigerous female of *P. grandis* was collected by a commercial trawler at depths of 220 m off northeastern Taiwan (24°52.352'N; 121°58.010'E). The berried female was reared in a 100 L aquarium and raised in sea water (salinity of 35) at 14 ± 1°C. Once the eggs hatched, approximately 400 actively swimming larvae were transferred to two beakers (5L). Each beaker contained similar number of larvae, with aerated seawater maintained at a temperature of 23 ± 1°C and a 12:12 hour photoperiod. Specimens of each zoeal stage were collected after the larvae moulted and preserved in a 70% ethylene glycol solution. At least two larvae from each stage were dissected and examined on glass slides under a stereo microscope (OLYMPUS SZX12) using fine entomological needles. Appendages were drawn using a *camera lucida* installed on a compound microscope (Olympus BX50). The descriptions and figures are arranged according to the standards proposed by Clark et al. (1998). Morphological terminology follows Yang and Ko (2004) and Landeira et al. (2010). Abbreviations of larval measurements are as follows: carapace length (**CL**), from the postorbital margin to the posteromedian end of the carapace; body length (**BL**), from the postorbital margin of the carapace to the posterior end of the telson; and total length (**TL**), from the tip of the rostrum to the tip of the telson. These are all given as mean values followed by the range (in parentheses). The female and larvae are deposited as vouchers in the National Taiwan Ocean University (NTOU M02079).

## Results

### Larval description

**Zoea I (Fig. 1)**

Period from hatching to the end of the instar: 1–8 days.

**Figure 1.**
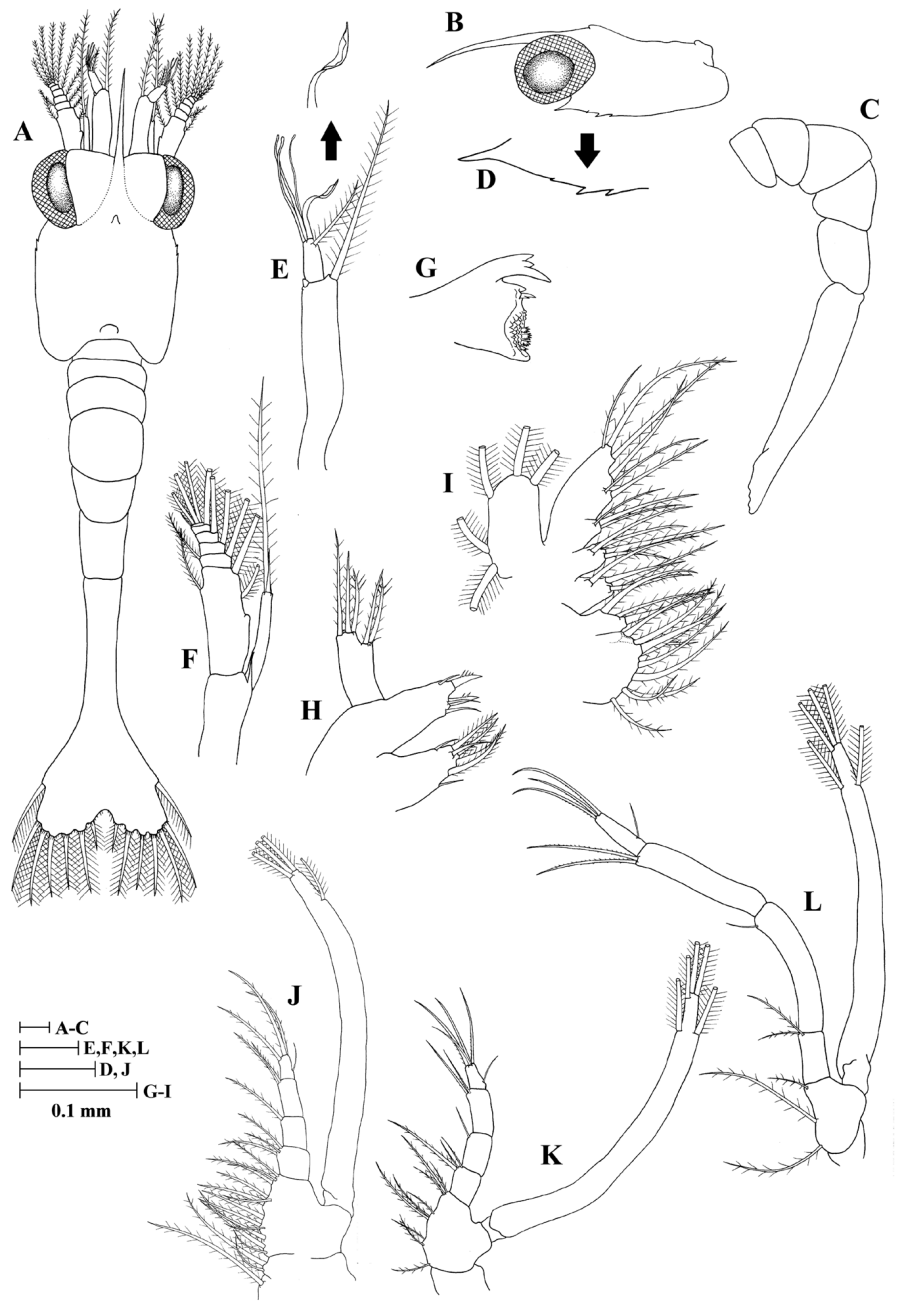
Zoea I of *Plesionika grandis*. **A** dorsal view **B** carapace lateral view **C** pleon lateral view **D** anteroventral margin of carapace **E** antennule **F** antenna **G** mandible **H** maxillule **I** maxilla **J** first maxilliped **K** second maxilliped **L** third maxilliped.

*Size* (n = 5): CL, 0.40 mm (0.38–0.42 mm); BL, 2.28 mm (2.27–2.30 mm); TL, 2.87 mm (2.82–2.90 mm).

*Carapace* (Fig. 1A, B, D) dorsoventrally flattened; rostrum slightly curved and slender, longer than antennular peduncle; dorsal anterior and posterior processes present; anteroventral margin bearing one strong pterygostomian spine and three unequal spines; eyes sessile.

*Antennule* (Fig. 1E) peduncle unsegmented, slender, and bearing one small tubercle; endopod with one long, plumose seta; exopod unsegmented with a single spatulate seta, three aesthetascs, and one distolateral seta.

*Antenna* (Fig. 1F) peduncle unsegmented with a sharp, basal spine distally; endopod unsegmented, with one long terminal, plumose seta, and a single sharp, slender spine distomesially; exopod 6-segmented, with eleven marginal plumose setae (3+2+1+1+1+3), proximal segment with one inner mesial tubercle, distal segment with one lateral simple seta.

*Mandible* (Fig. 1G) palp absent; incisor with three terminal teeth; lacinia mobilis present.

*Maxillule* (Fig. 1H) coxal endite with seven (two simple subterminal + five terminal plumose) setae; basial endite with two strong, cuspidate setae and three simple setae; endopod unsegmented, 3-lobed with one small, simple seta and two sparsely plumose setae on basal lobe, two sparsely plumose setae on median lobe, and one sparsely plumose setae on distal lobe; exopod absent.

*Maxilla* (Fig. 1I) coxal endite bilobed with 9 + 4 plumose setae; basal endite bilobed with 4 + 4 plumose setae; endopod with nine (3 + 2 + 1 + 1 + 2) setae; scaphognathite margin with five plumose setae.

*First maxilliped* (Fig. 1J) coxa with three plumose setae; basis with 12 plumose setae; endopod 4-segmented with three, one, two, four (one outer + three terminal) setae; exopod unsegmented, armed distally with four plumose, natatory setae.

*Second maxilliped* (Fig. 1K) coxa without setae; basis with nine plumose setae; endopod 4-segmented with three, one, two, five (one outer + four terminal) setae; exopod unsegmented, armed distally with five plumose, natatory setae.

*Third maxilliped* (Fig. 1L) coxa without setae; basis with three plumose setae; endopod 4-segmented with two, one, two, four (one outer + three terminal) setae; exopod unsegmented, armed distally with five plumose, natatory setae.

*Pereiopods* absent.

*Pleon* (Fig. 1A, C) with five somites, no spines or setae.

*Pleopods* absent.

*Uropods* absent.

*Telson* (Fig. 1A) subtriangular, posterior margin minutely spinulated except on distolateral parts, with 7 + 7 plumose setae, outermost two pairs only plumose on inner margin.

**Figure 2.**
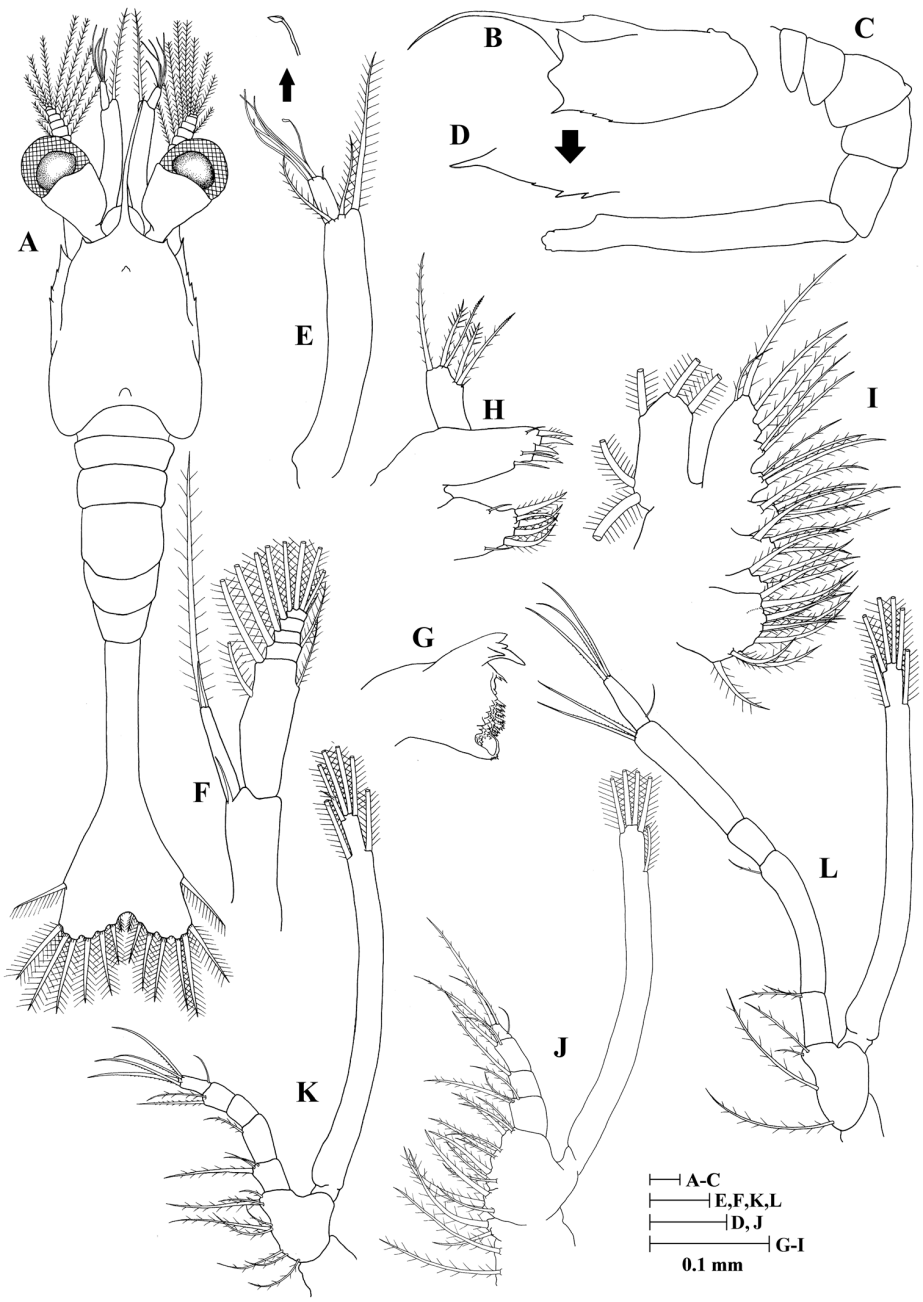
Zoea II of *Plesionika grandis*. **A** dorsal view **B** carapace lateral view **C** pleon lateral view **D** anteroventral margin of carapace **E** antennule **F** antenna **G** mandible **H** maxillule **I** maxilla **J** first maxilliped **K** second maxilliped **L** third maxilliped.

**Zoea II (Fig. 2)**

Period from hatching to the end of the instar: 8–12 days.

*Size* (n = 4): CL, 0.45 mm (0.43–0.48 mm); BL, 2.43 mm (2.41–2.44 mm); TL, 2.98 mm (2.96–3.01 mm).

*Carapace* (Fig. 2A, B, D) rostrum curved and slender, longer than antennular peduncle, nearly as long as carapace length; supraorbital spine present; eyes stalked, funnel-shaped; other unchanged.

*Antennule* (Fig. 2E) peduncle unsegmented, bearing two terminal plumose setae; endopod with one long, plumose seta; exopod unsegmented with one spatulate seta, four aesthetascs and one simple seta.

*Antenna* (Fig. 2F) unchanged.

*Mandible* (Fig. 2G) palp absent; incisor with three terminal and one subterminal teeth; lacinia mobilis serrate.

*Maxillule* (Fig. 2H) basial endite with four strong cuspidate setae and three simple setae; other unchanged.

*Maxilla* (Fig. 2I) coxal endite bilobed with 10 + 4 plumose setae; other unchanged.

*First maxilliped* (Fig. 2J) coxa with four plumose setae; exopod unsegmented, armed distally with five plumose, natatory setae; other unchanged.

*Second maxilliped* (Fig. 2K) endopod 5-segmented with three, one, zero, two, five (one outer + four terminal) setae; exopod unsegmented, armed distally with six plumose, natatory setae; other unchanged.

*Third maxilliped* (Fig. 2L) coxa without setae; basis with four plumose setae; endopod 5-segmented with two, one, zero, two, four (one outer + three terminal) setae; exopod unsegmented, armed distally with six plumose, natatory setae.

*Pereiopods* absent.

*Pleon* (Fig. 2A, C) unchanged.

*Pleopods* absent.

*Uropod* absent.

*Telson* (Fig. 2A) subtriangular, posterior margin with 8 + 8 plumose setae, only outermost pair plumose on inner margin; other unchanged.

**Figure 3.**
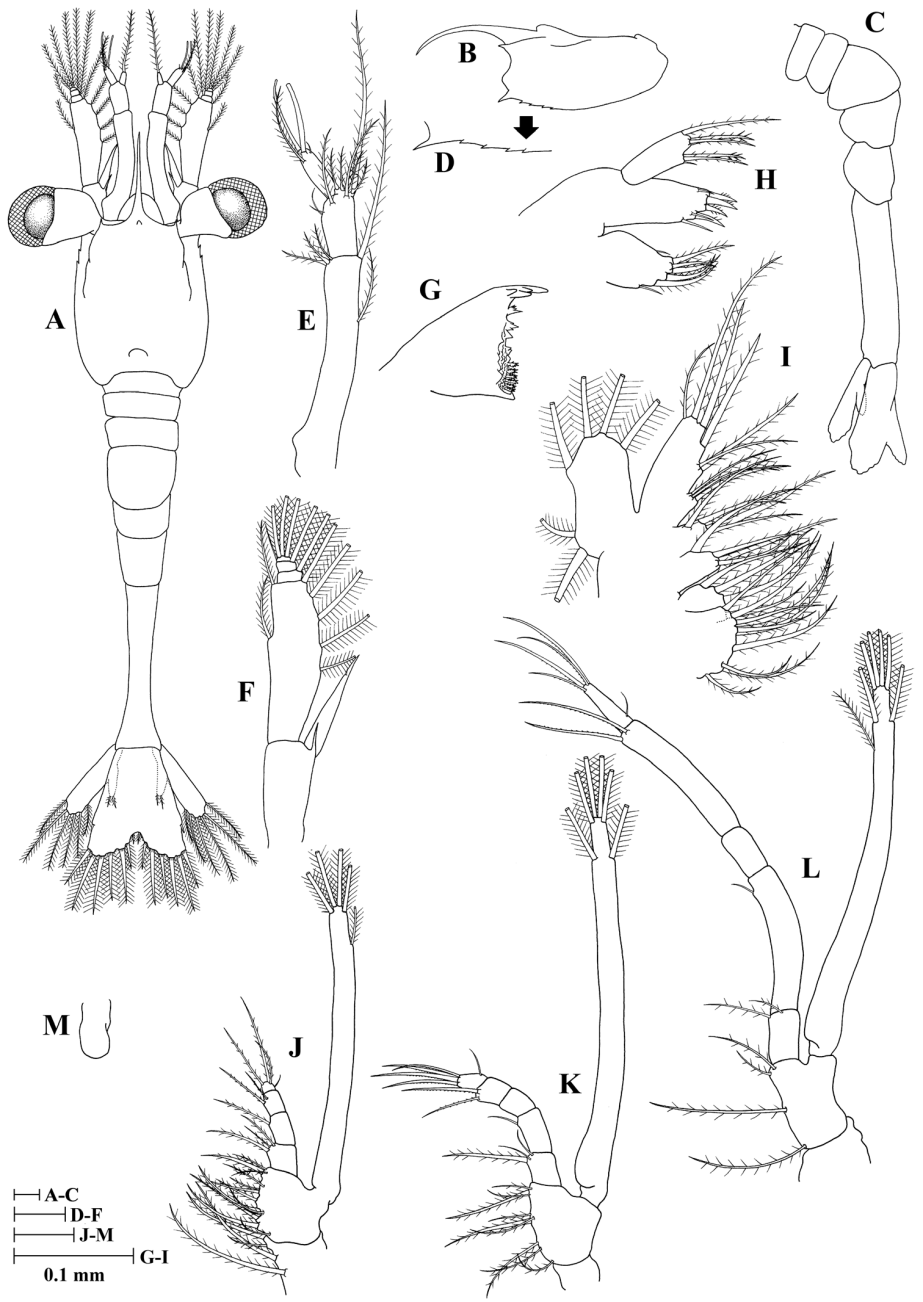
Zoea III of *Plesionika grandis*. **A** dorsal view **B** carapace lateral view **C** pleon lateral view **D** anteroventral margin of carapace **E** antennule **F** antenna **G** mandible **H** maxillule **I** maxilla **J** first maxilliped **K** second maxilliped **L** third maxilliped **M** first pereiopod.

**Zoea III (Fig. 3)**

Period from hatching to the end of the instar: 12–17 days.

Size (n = 3): CL, 0.52 mm (0.50–0.55 mm); BL, 2.45 mm (2.39–2.53 mm); TL, 2.94 mm (2.81–3.08 mm).

*Carapace* (Fig. 3A, B, D) rostrum shorter than in previous stages but still curved, 0.85 times as long as carapace length; ventrolateral margin with four spines posterior to pterygostomial spine; other unchanged.

*Antennule* (Fig. 3E) peduncle 2-segmented: basal segment with two long and three short plumose setae; distal segment with two groups of setae, one consisting of six plumose and other with two simple setae; endopod with one long plumose seta; exopod unsegmented with two aesthetasc, one plumose and one simple setae.

*Antenna* (Fig. 3F) peduncle unchanged; endopod unsegmented, with one spiniform seta and one simple short seta; exopod distally 4-segmented, with 12 plumose setae and one distolateral seta.

*Mandible* (Fig. 3G) unchanged.

*Maxillule* (Fig. 3H) unchanged.

*Maxilla* (Fig. 3I) scaphognathite margin with six plumose setae; other unchanged.

*First maxilliped* (Fig. 3J) unchanged.

*Second maxilliped* (Fig. 3K) unchanged.

*Third maxilliped* (Fig. 3L) endopod 5-segmented with 2, 1, 0, 2, 5 (one outer + four terminal) setae; exopod unsegmented, armed distally with seven plumose natatory setae; other unchanged.

*Pereiopods* (Fig. 3M) first pereiopod as bud; second to fifth pereiopods absent.

*Pleon* (Fig. 3A, C) with six somites; other unchanged.

*Pleopods* absent.

*Uropod* (Fig. 3A) biramous. Endopod rudimentary with two plumose setae; exopod well developed with six plumose setae.

*Telson* (Fig. 3A) with seven pairs of terminal plumose setae and one pair of outermost short, simple, subterminal setae.

**Figure 4.**
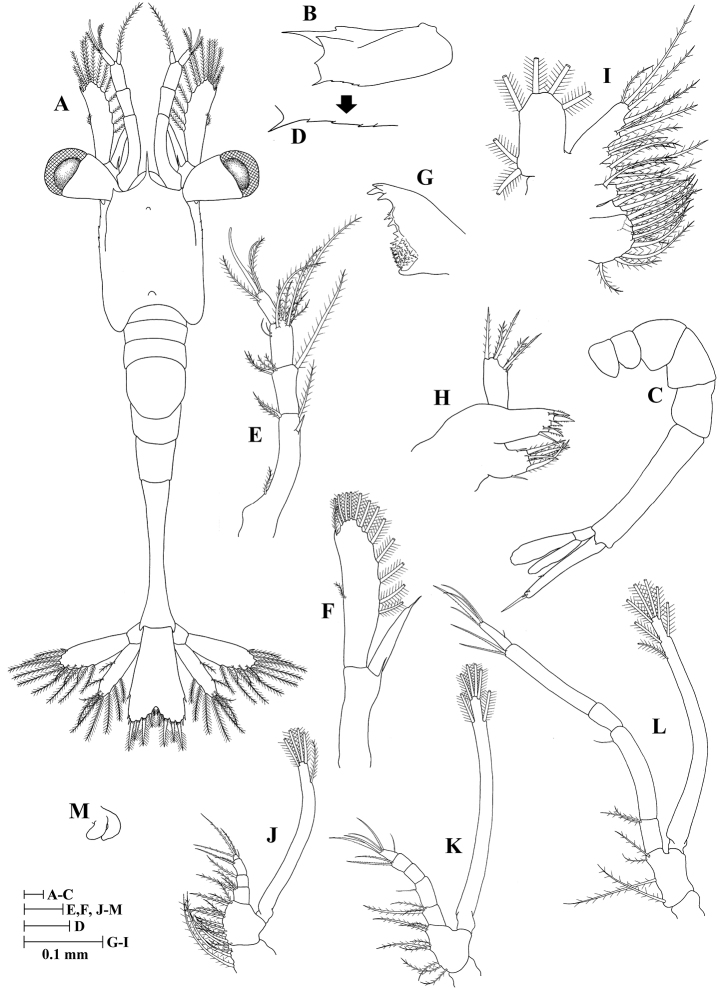
Zoea IV of *Plesionika grandis*, **A** dorsal view **B** carapace lateral view **C** pleon lateral view **D** anteroventral margin of carapace **E** antennule **F** antenna **G** mandible **H** maxillule **I** maxilla **J** first maxilliped **K** second maxilliped **L** third maxilliped **M** first pereiopod.

**Zoea IV (Fig. 4)**

Period from hatching to the end of the instar: 17–21 days.

Size (n = 2): CL, 0.56 mm (0.54–0.58 mm); BL, 3.03 mm (2.98–3.07 mm); TL, 3.26 mm (3.25–3.26 mm).

*Carapace* (Fig. 4A, B, D) rostrum not curved and shorter than in previous stages but longer than frontal lobe, 0.35 times as long as carapace length; other unchanged.

*Antennule* (Fig. 4E) peduncle 3-segmented: basal segment with four (one proximal, one terminal long and two terminal short) plumose setae plus one subterminal strong spiniform seta; medial segment with five (one long, four short) plumose setae; distal segment with two groups of setae, one consisting of six plumose (two subterminal + four terminal) setae and other with two simple setae; endopod unchanged; exopod unsegmented with two aesthetascs and two plumose setae.

*Antenna* (Fig. 4F) endopod unsegmented with one spiniform seta; exopod unsegmented with one apical spine, 13 plumose setae on inner margin, and one plumose seta on outer margin; other unchanged.

*Mandible* (Fig. 4G) incisor having four teeth; molar process with numerous small teeth; other unchanged.

*Maxillule* (Fig. 4H) unchanged.

*Maxilla* (Fig. 4I) unchanged.

*First maxilliped* (Fig. 4J) unchanged.

*Second maxilliped* (Fig. 4K) unchanged.

*Third maxilliped* (Fig. 4L) unchanged.

*Pereiopods* (Fig. 4M) first pereiopod as biramous bud; second to fifth pereiopods absent.

*Pleon* (Fig. 4A, C) unchanged.

*Pleopods* absent.

*Uropod* (Fig. 4A, C) protopod without setae; endopod well developed with ten plumose setae; exopod with 12 plumose setae and one simple seta at outermost apex.

*Telson* (Fig. 4A) less triangular than in zoea III; one lateral simple seta, posterior margin with five pairs of plumoserrulate setae, and two outer simple setae on each side.

**Zoea V (Fig. 5)**

Period from hatching to the end of the instar: 21–23 days.

**Figure 5.**
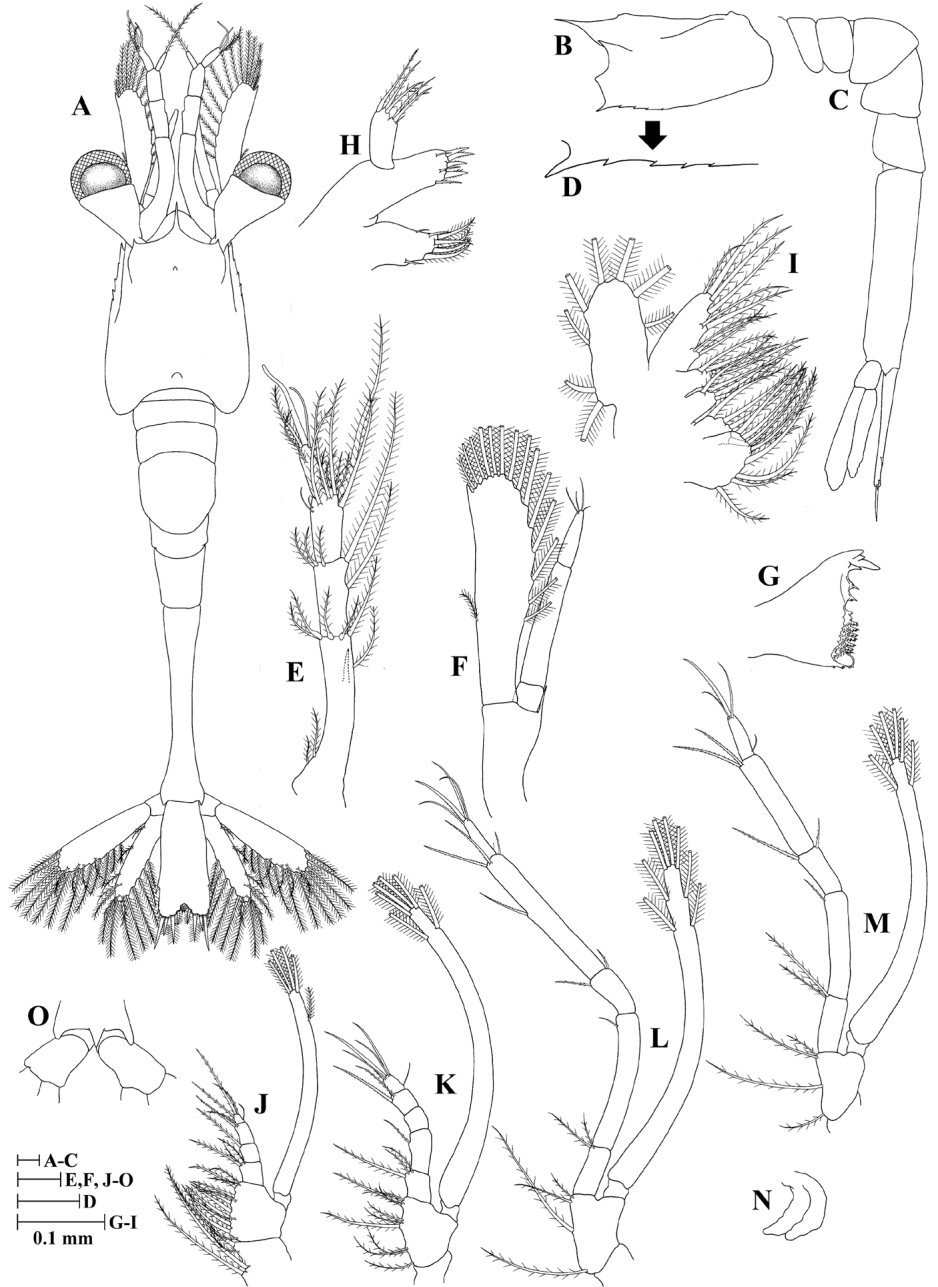
Zoea V of *Plesionika grandis*, **A** dorsal view **B** carapace lateral view **C** pleon lateral view **D** anteroventral margin of carapace **E** antennule **F** antenna **G** mandible **H** maxillule **I** maxilla **J** first maxilliped **K** second maxilliped **L** third maxilliped **M** first pereiopod **N** second pereiopod **O** ventral view of anal spine.

Size (n = 3): CL, 0.61 mm (0.59–0.62 mm); BL, 3.05 mm (3.00–3.11 mm); TL, 3.25 mm (3.22–3.29 mm).

*Carapace* (Fig. 5A, B, D) unchanged.

*Antennule* (Fig. 5E) peduncle 3-segmented: basal segment with eight (two proximal, one subterminal, one terminal long, four terminal short) plumose seta plus one subterminal strong spiniform seta; medial segment with five (one subterminal long, one terminal long, three terminal short) plumose setae; distal segment with two groups of setae, one consisting of eight (four subterminal + four terminal) plumose setae and other with two simple setae; endopod unchanged; exopod with two aesthetascs and two plumose setae.

*Antenna* (Fig. 5F) endopod 3-segmented, with 0, 0, 4 simple setae; exopod unsegmented with apical spine, 14 plumose setae on inner margin and one plumose seta on outer margin; other unchanged.

*Mandible* (Fig. 5G) unchanged.

*Maxillule* (Fig. 5H) coxal endite with eight (three simple, subterminal + five terminal, plumose) setae; other unchanged.

*Maxilla* (Fig. 5I) scaphognathite margin with eight plumose setae; other unchanged.

*First maxilliped* (Fig. 5J) unchanged.

*Second maxilliped* (Fig. 5K) unchanged.

*Third maxilliped* (Fig. 5L) endopod 5-segmented with two, one, two (one inner + one outer), four (three inner + one outer), and five (one outer + four terminal) setae; exopod unsegmented, armed distally with eight plumose natatory setae; other unchanged.

*First pereiopod* (Fig. 5M) coxa without setae; basis with four plumose setae; endopod 5-segmented with two, one, two (one inner + one outer), two, four (one outer + three terminal) setae; exopod unsegmented, armed distally with six long, plumose natatory setae.

*Second pereiopod* (Fig. 5N) as biramous bud.

*Third, fourth, and fifth pereiopods* absent.

*Pleon* (Fig. 5A, C, O) anal spine present; shallow notch on lateral margin of fourth pleomere; other unchanged.

*Pleopods* absent.

*Uropod* (Fig. 5A, C) protopod unchanged; endopod with 15 plumose setae; exopod with 16 plumose setae plus one simple seta at outermost apex.

*Telson* (Fig. 5A) almost rectangular; other unchanged.

**Figure 6.**
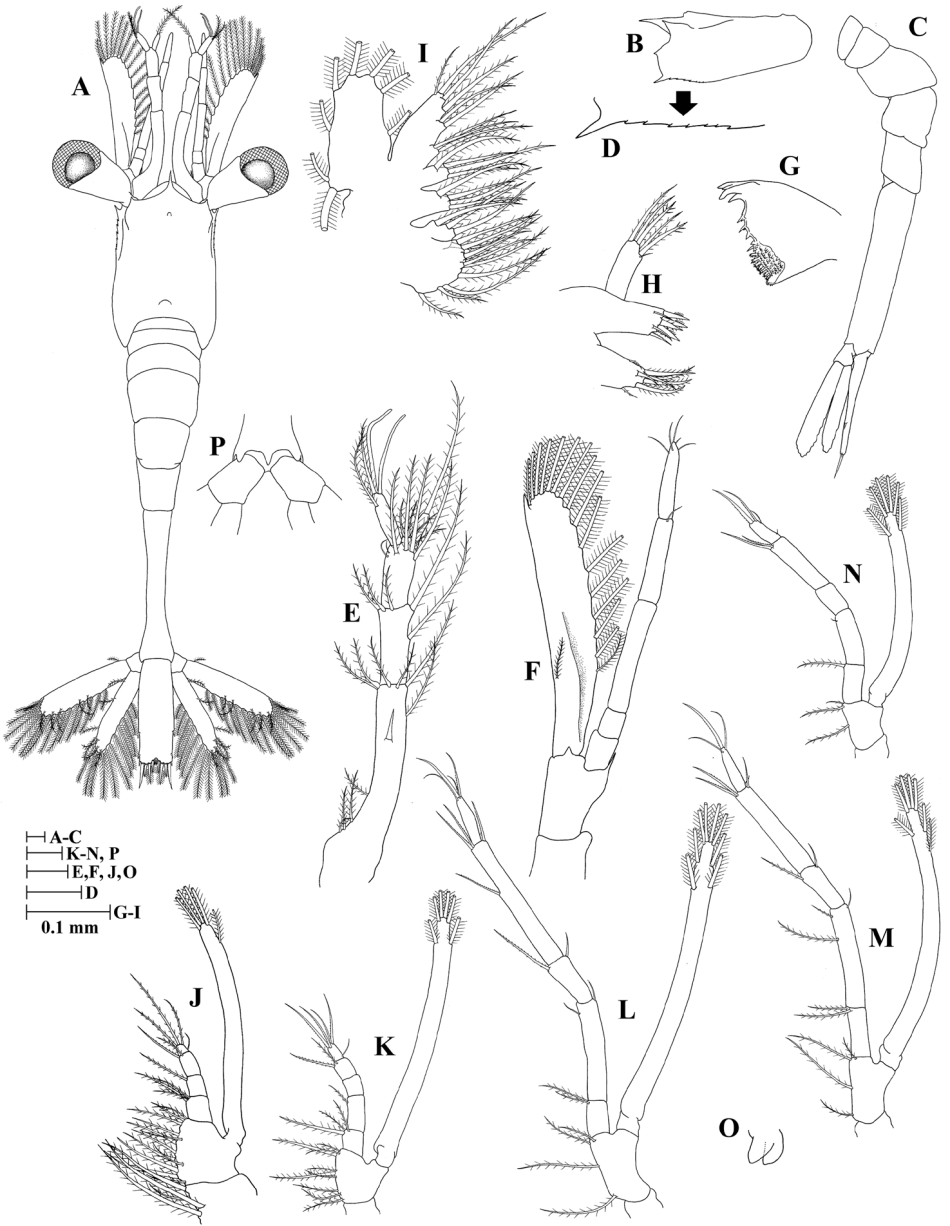
Zoea VI of *Plesionika grandis*, **A** dorsal view **B** carapace lateral view **C** pleon lateral view **D** anteroventral margin of carapace **E** antennule **F** antenna **G** mandible **H** maxillule **I** maxilla **J** first maxilliped **K** second maxilliped **L** third maxilliped **M** first pereiopod **N** second pereiopod **O** third pereiopod **P** ventral view of anal spine.

**Zoea VI (Fig. 6)**

Period from hatching to the end of the instar: 23–29 days.

Size (n = 2): CL, 0.62 mm (0.60–0.65 mm); BL, 3.07 mm (3.04–3.10 mm); TL, 3.26 mm (3.22–3.30 mm).

*Carapace* (Fig. 6A, B, D) rostrum short 0.25 times as long as carapace length; pterygostomian spine present; ventrolateral margin with seven spines posterior to pterygostomial spine; other unchanged.

*Antennule* (Fig. 6E) peduncle 3-segmented, basal segment with four proximal plumose setae; other unchanged.

*Antenna* (Fig. 6F) peduncle 2-segmented, distal segment with two basal spines; endopod five-segmented with zero, zero, zero, one, five simple setae; exopod unsegmented with apical spine, 16 plumose setae on inner margin, and one plumose seta on lateral margin.

*Mandible* (Fig. 6G) unchanged.

*Maxillule* (Fig. 6H) coxal endite with eight plumose setae (three subterminal + five terminal); other unchanged.

*Maxilla* (Fig. 6I) unchanged.

*First maxilliped* (Fig. 6J) unchanged.

*Second maxilliped* (Fig. 6K) unchanged.

*Third maxilliped* (Fig. 6L) endopod 5-segmented with two, two (one inner + one outer), two (one inner + one outer), four (three inner + one outer), and five (one outer + four terminal) setae; other unchanged.

*First pereiopod* (Fig. 6M) endopod 5-segmented with two, three (two inner + one outer), two (one inner + one outer), three (two inner + one outer), and four (one outer + three terminal) setae; exopod unsegmented, armed distally with seven plumose natatory setae; other unchanged.

*Second pereiopod* (Fig. 6N) coxa without setae, basis with three plumose setae; endopod 5-segmented with one, one (short), zero, two, four (one outer + three terminal) setae; exopod unsegmented, armed distally with six plumose natatory setae.

*Third pereiopod* (Fig. 6O) as biramous bud.

*Fourth and fifth pereiopods* absent.

*Pleon* (Fig. 6A, C, P) unchanged.

*Pleopods* absent.

*Uropod* (Fig. 6A, C) endopod well developed with 18 plumose setae; exopod with 20 plumose setae plus one plumose seta on outer margin, and one simple seta at outermost apex; other unchanged.

*Telson* (Fig. 6A) rectangular; other unchanged.

**Figure 7.**
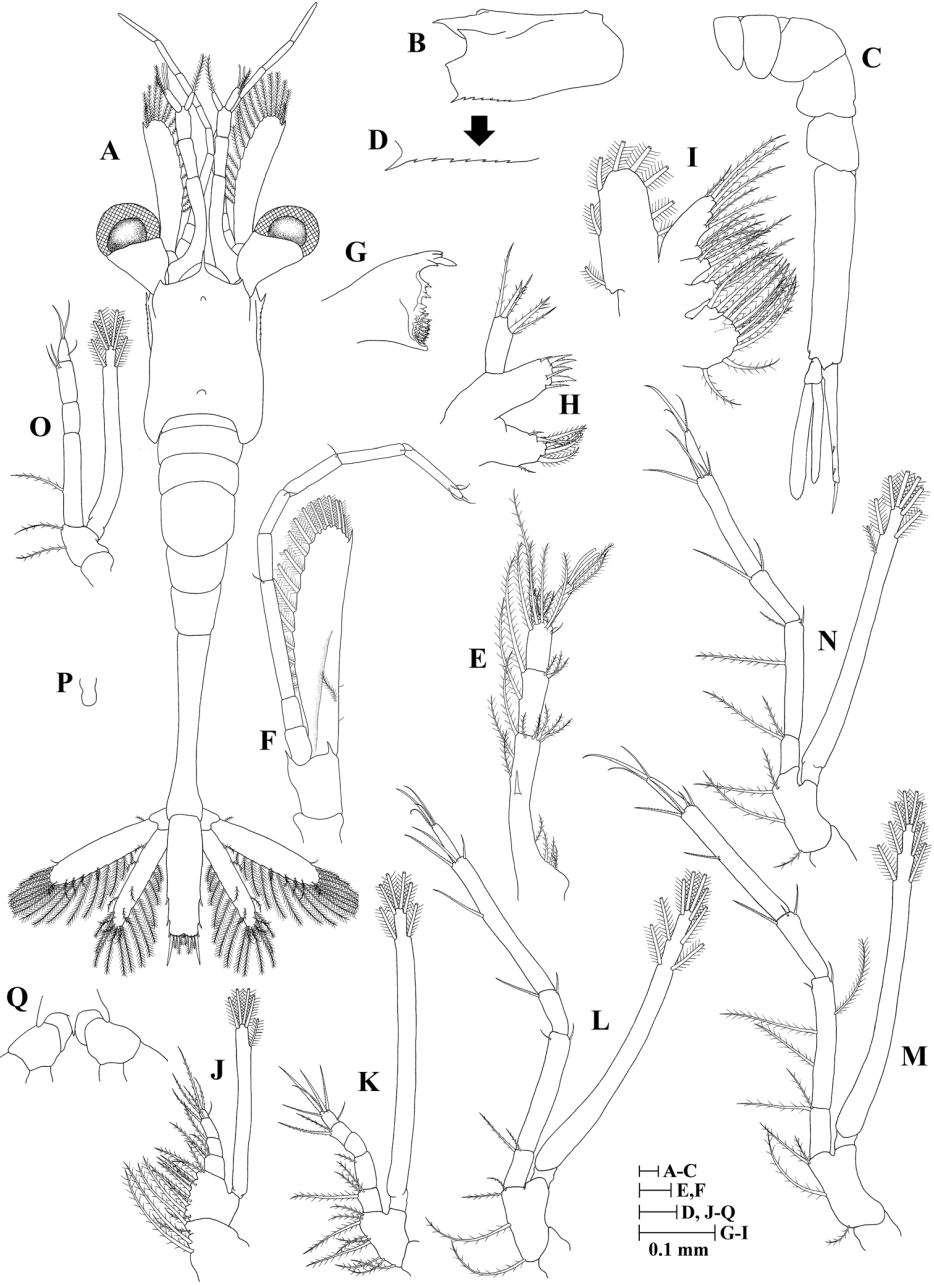
Zoea VII of *Plesionika grandis*, **A** dorsal view **B** carapace lateral view **C** pleon lateral view **D** anteroventral margin of carapace **E** antennule **F** antenna **G** mandible **H** maxillule **I** maxilla **J** first maxilliped **K** second maxilliped **L** third maxilliped **M** first pereiopod **N** second pereiopod **O** third pereiopod **P** fourth pereiopod **Q** ventral view of anal spine.

**Zoea VII (Fig. 7)**

Period from hatching to the end of the instar: 29–36 days.

Size (n = 2): CL, 0.68 mm (0.64–0.72 mm); BL, 3.75 mm (3.40–4.10 mm); TL, 3.98 mm (3.62–4.34 mm).

*Carapace* (Fig. 7A, B, D) unchanged.

*Antennule* (Fig. 7E) peduncle 3-segmented, basal segment with 13 (five proximal, two subterminal, one terminal long and five terminal short) plumose setae plus one subterminal strong spiniform seta; other unchanged.

*Antenna* (Fig. 7F) endopod 8-segmented with 0, 0, 1, 0, 1, 1, 1, 5 simple setae; exopod unsegmented with one apical spine, 17 plumose setae on inner margin, outer margin with one plumose and two simple setae; others unchanged.

*Mandible* (Fig. 7G) unchanged.

*Maxillule* (Fig. 7H) unchanged.

*Maxilla* (Fig. 7I) unchanged.

*First maxilliped* (Fig. 7J) unchanged.

*Second maxilliped* (Fig. 7K) endopod 5-segmented with three, one, zero, two, six (one outer + five terminal) setae; other unchanged.

*Third maxilliped* (Fig. 7L) endopod 5-segmented with two, two (one inner + one outer), two (one inner + one outer), four (three inner + one outer), and four (terminal, no outer) setae; other unchanged.

*First pereiopod* (Fig. 7M) endopod 5-segmented with two, five (inner with two plumose, one simple; outer with one plumose, one simple), three (one inner + one lateral + one outer), five (three inner + two outer), and three (terminal, no outer) setae; exopod unsegmented, armed distally with eight plumose natatory setae; other unchanged.

*Second pereiopod* (Fig. 7N) coxa without setae, basis with four plumose setae; endopod 5-segmented with two, three (two inner + one outer), two (one inner + one outer), four (two inner + two outer), and four (one outer + three terminal) setae; exopod unsegmented, armed distally with seven plumose natatory setae.

*Third pereiopod* (Fig. 7O) coxa without setae; basis with two plumose setae; endopod 4-segmented with one, zero, two, three (one outer + two terminal) setae; exopod unsegmented, armed distally with six plumose natatory setae.

*Fourth pereiopod* (Fig. 7P) as bud.

*Fifth pereiopod* absent.

*Pleon* (Fig. 7A, C, Q) unchanged.

*Pleopods* absent.

*Uropod* (Fig. 7A, C) endopod with 22 plumose setae; exopod with 21 plumose setae plus one plumose and one simple setae at outer margin, one simple seta at outermost apex; others unchanged.

*Telson* (Fig. 7A) unchanged.

**Figure 8.**
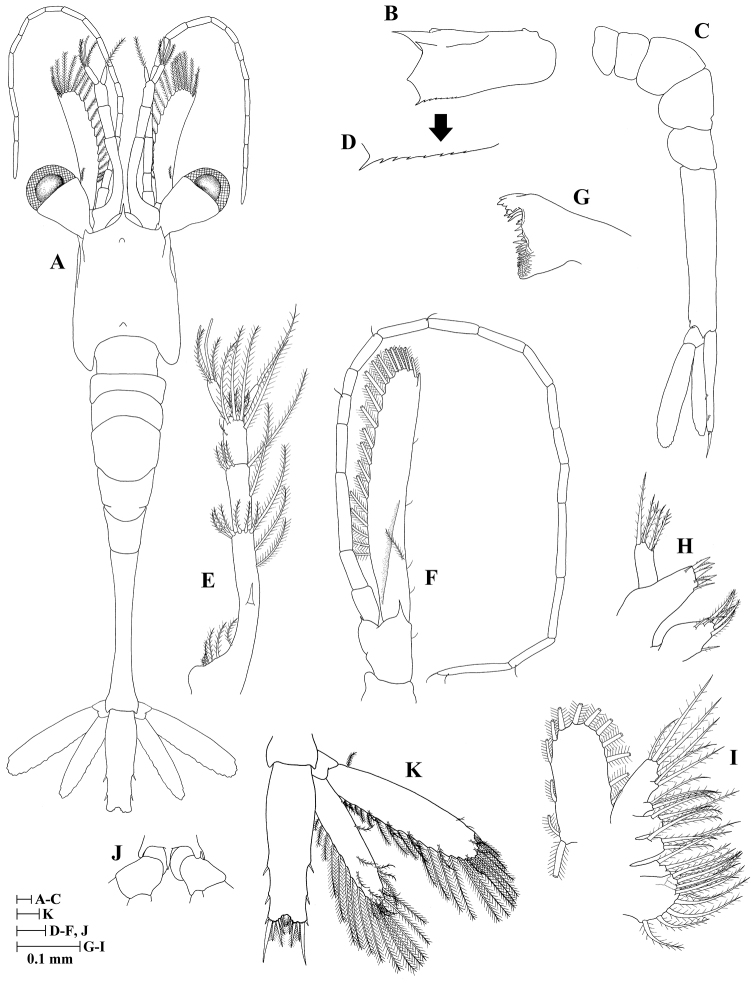
Zoea VIII of *Plesionika grandis*, **A** dorsal view **B** carapace lateral view **C** pleon lateral view **D** anteroventral margin of carapace **E** antennule **F** antenna **G** mandible **H** maxillule **I** maxilla **J** ventral view of anal spine **K** uropod and telson.

**Figure 9.**
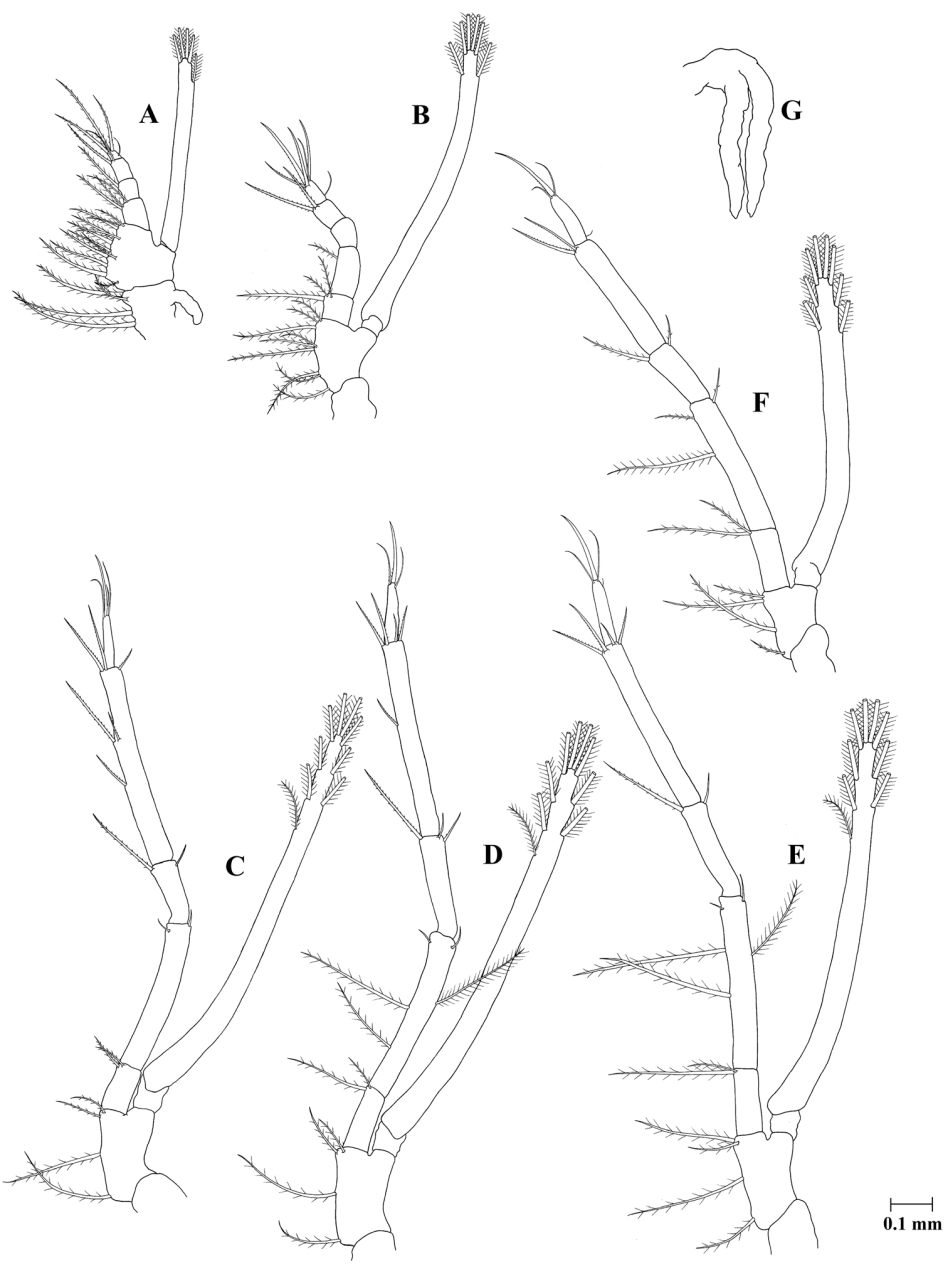
Zoea VIII of *Plesionika grandis*, **A** first maxilliped **B** second maxilliped **C** third maxilliped **D** first pereiopod **E** second pereiopod **F** third pereiopod **G** fourth pereiopod.

**Zoea VIII (Figs 8, 9)**

Period from hatching to the end of the instar: 36 days.

Size (n = 3): CL, 0.84 mm (0.80–0.92 mm); BL, 4.16 mm (3.88–4.37 mm); TL, 4.36 mm (4.06–4.55 mm).

*Carapace* (Fig. 8A, B, D) unchanged.

*Antennule* (Fig. 8E) peduncle 3-segmented: basal segment with 15 (six proximal, two subterminal, one terminal long, six short) plumose setae plus one subterminal strong spiniform seta; other unchanged.

*Antenna* (Fig. 8F) peduncle with one simple seta on outer margin; endopod 18-segmented with 0, 0, 0, 0, 1, 0, 1, 1, 0, 0, 0, 0, 0, 0, 0, 0,1, 2 simple setae; exopod unsegmented with one apical spine, 20 plumose setae on inner margin, outer margin with one plumose and four simple setae; other unchanged.

*Mandible* (Fig. 8G) incisor with four or more terminal teeth; molar process with numerous small teeth; other unchanged.

*Maxillule* (Fig. 8H) unchanged.

*Maxilla* (Fig. 8I) scaphognathite margin with 13 plumose setae; other unchanged.

*First maxilliped* (Fig. 9A) epipod newly appeared; other unchanged.

*Second maxilliped* (Fig. 9B) unchanged.

*Third maxilliped* (Fig. 9C) endopod 5-segmented with two, two (one inner + one outer), two (one inner + one outer), six (five inner + one outer), and four (terminal) setae; exopod unsegmented, armed distally with nine plumose, natatory setae; other unchanged.

*First pereiopod* (Fig. 9D) exopod unsegmented, armed distally with nine plumose natatory setae; other unchanged.

*Second pereiopod* (Fig. 9E) endopod 5-segmented with two, five (inner with two plumose, one simple; outer with one plumose, one simple), two (one inner + one outer), four (two inner + two outer), and four (terminal) setae; exopod unsegmented, armed distally with nine plumose, natatory setae; other unchanged.

*Third pereiopod* (Fig. 9F) coxa unchanged; basis with four setae; endopod 5-segmented with two, three (two inner + one outer), two (one inner + one outer), two, four (one outer + three terminal) setae; exopod unsegmented, armed distally with eight plumose natatory setae.

*Fourth pereiopod* (Fig. 9G) as biramous bud.

*Fifth pereiopod* absent.

*Pleon* (Fig. 8A, C, J) unchanged.

*Pleopods* absent.

*Uropod* (Fig. 8K) endopod with 27 plumose setae; exopod with 28 plumose setae plus one plumose, three simple setae on outer margin, one simple seta at outermost apex; other unchanged.

*Telson* (Fig. 8A, K) less rectangular, shaped like inverted triangle, other unchanged.

## Discussion

The first eight zoeal stages of *Plesionika grandis* were obtained in 36 days after hatching, representing the longest larval rearing record for the genus. Previous longest larval culture for *Plesionika* shrimps was *P. edwardsii* by Landeira et al. 2009a, lasting 20 days, and reaching the Z7 stage. It is suspected that feeding and/or rearing temperature may be the main causes for the mortality of the larvae as discussed in our previous work on the larval rearing of another deep-sea pandalid shrimp *Heterocarpus abulbus* Yang, Chan and Chu, 2010 (Jiang et al. 2016).

Features of each larval stage as well as changes in appendage setation and setal types in *P. grandis* are summarized in Table 1. The major characters of each zoeal stage are: (ZI) sessile eyes, three pairs of maxillipeds, pleon with five somites and telson subtriangular; (ZII) eyes stalked; (ZIII) uropod with exopod well developed, first pereiopod appeared, and pleon with six somites; (ZIV) antennular peduncle segmented, uropod with endopod; (ZV) endopod of antenna 3-segmented, second pereiopod appeared, and anal spine present on sixth abdominal somite; (ZVI) third pereiopod appeared, telson becoming rectangular; (ZVII) endopod of antenna with more than three segments, and fourth pereiopod appeared as a bud; (ZVIII) endopod of antenna with more than ten segments, and fourth pereiopod biramous.

**Table 1.** Characteristics of the zoeal stage of *Plesionika grandis*. a, aesthetasc; b, basal spine; c, cuspidate seta; d, distolateral seta; s, simple seta; spi, spiniform seta; p, plumose seta; spa, spatulate seta; st, strong seta; sl, slender spine.

	Z1	Z2	Z3	Z4	Z5	Z6	Z7	Z8
**Duration (days)**	1-8	8-12	12-17	17-21	21-23	23-29	29-36	36
**Carapace length (mm)**	0.40	0.45	0.52	0.56	0.61	0.62	0.68	0.84
**Anterolateral spines**	3	3	4	4	4	7	7	7
**Antennule**								
Peduncle	1 tubercle	2p	5p,6p+2s	1spi+4p,5p,6p+2s	1spi+8p,5p,8p+2s	1spi+10p,5p,8p+2s	1spi+13p,5p,8p+2s	1spi+15p,5p,8p+2s
Endopod	1d	1p	1p	1p	1p	1p	1p	1p
Exopod	3a+1d+1s	4a+1s+1spa	2a+1p+1s	2a+2p	2a+2p	2a+2p	2a+2p	2a+2p
**Antenna**								
Peduncle	1b	1b	1b	1b	1b	2b	2b	2b
Endopod	1p+1sl	1p+1sl	1spi+1s	1spi	0,0,4	0,0,0,1,5	0,0,1,0,1,1,1,5	0,0,0,0,1,0,1,1,0,0,0,0,0,0,0,0,1,2
Exopod	11p+1d	11p+1d	12p+1d	13p+1p	14p+1p	16p+1p	17p+1p+2s	20p+1p+4s
**Maxillule**								
Coxal endite setation	2s+5p	2s+5p	2s+5p	2s+5p	3s+5p	8p	8p	8p
Basial endite setation	2st+3c	4st+3c	4st+3c	4st+3c	4st+3c	4st+3c	4st+3c	4st+3c
Endopod setation	1s+5p	1s+5p	1s+5p	1s+5p	1s+5p	1s+5p	1s+5p	1s+5p
Exopod setation	0	0	0	0	0	0	0	0
**Maxilla**								
Coxal endite setation	9p+4p	10p+4p	10p+4p	10p+4p	10p+4p	10p+4p	10p+4p	10p+4p
Basial endite setation	4p+4p	4p+4p	4p+4p	4p+4p	4p+4p	4p+4p	4p+4p	4p+4p
Endopod setation	3+2+1+1+2	3+2+1+1+2	3+2+1+1+2	3+2+1+1+2	3+2+1+1+2	3+2+1+1+2	3+2+1+1+2	3+2+1+1+2
Exopod setation	5p	5p	6p	6p	8p	8p	8p	13p
**First Maxilliped**								
Coxal endite setation	3p	4p	4p	4p	4p	4p	4p	4p
Basis endite setation	12p	12p	12p	12p	12p	12p	12p	12p
Endopod setation	3,1,2,4	3,1,2,4	3,1,2,4	3,1,2,4	3,1,2,4	3,1,2,4	3,1,2,4	3,1,2,4
Exopod setation	4p	5p	5p	5p	5p	5p	5p	5p
**Second Maxilliped**								
Coxal endite setation	0	0	0	0	0	0	0	0
Basis endite setation	9p	9p	9p	9p	9p	9p	9p	9p
Endopod setation	3,1,2,5	3,1,0,2,5	3,1,0,2,5	3,1,0,2,5	3,1,0,2,5	3,1,0,2,5	3,1,0,2,6	3,1,0,2,6
Exopod setation	5p	6p	6p	6p	6p	6p	6p	6p
**Third Maxilliped**								
Coxal endite setation	0	0	0	0	0	0	0	0
Basis endite setation	3p	4p	4p	4p	4p	4p	4p	4p
Endopod setation	2,1,2,4	2,1,0,2,4	2,1,0,2,5	2,1,0,2,5	2,1,2,4,5	2,2,2,4,5	2,2,2,4,4	2,2,2,6,4
Exopod setation	5p	6p	7p	7p	8p	8p	8p	9p

The early zoeal morphology of *P. grandis* has the common characters of pandalid larvae, such as eye peduncle narrowed at base, carapace with two dorsal protuberances and anteroventral margin bearing spines, antennule with peduncle strongly concave and exopod bearing spatulate seta, antenna with segmented exopod, rostrum elongated in earlier stages (see Thatje and Bacardit 2000; Landeira et al. 2010; Jiang et al. 2014). Only two species of *Plesionika* have their larvae reported. They are *P. edwardsii* by Landeira et al. 2009a [ZI-ZVII] and *P. narval* by Landeira et al. 2009b, 2014 [ZI-ZV, decapodid]. The zoeae of these three species mainly differ in the following characters:

(1) Number of spines on anteroventral margin of carapace: *P. edwardsii* with two spines in ZI, but disappeared in ZII and later stages; *P. narval* with three spines in ZI to ZV; *P. grandis* with three spines in ZI, increased to four spines in ZIII, and then, seven spines in ZVI.

(2) Number of tubercles on antennule in ZI: *P. edwardsii* and *P. narval* with two tubercles, *P. grandis* with one.

(3) Endopod segmentation of antenna: *P. grandis* 3-segmented in ZV, 5-segmented in ZVI, 8-segmented in ZVII, 18-segmented in ZVIII; *P. edwardsii* segmented only in ZVII and 3-segmented; *P. narval* segmented since ZV and 2-segmented.

(4) Third maxilliped setation in ZI: Basis with three setae in *P. edwardsii* and *P. grandis*, whilst *P. narval* with four setae. Moreoever, *P. edwardsii* has a somewhat different endopod setation at the third maxilliped (1, 1, 2, 4 *vs.* 2, 1, 2, 4 in the other two species).

Furthermore, the ZVII of *P. grandis* appeared to be more developed than that of *P. edwardsii* by having the first three pairs of pereiopods well developed (*vs.* only first two pereiopods well developed in the latter). This indicates that the larval development of *P. edwardsii* may have even longer duration. Although the numerous species in *Plesionika* are often separated into species groups (see Chan and Crosnier 1991, 1997; Chan 2004) with *P. narval* and *P. grandis* belonging to the same species group, their early larval stages are not more similar to each other than to *P. edwardsii*.

Species of *Plesionika* likely have very long larval development (see Landeira et al. 2014). Compared to the long larval development in other caridean shrimps such as *Rhynchocinetes conspiciocellus* Okuno & Takeda, 1992 (eleven zoeal stages to decapodid in 112 days, Matoba and Shokita 1998) and *Macrobrachium lar* (Fabricius, 1798) (eight zoeal stages to decapodid in 110 days, Lal et al. 2014), their pleopods only firstly appeared at three stages before the final zoeal stage (i.e. *R. conspiciocellus* in ZVIII; *M. lar* in ZV). Since the ZVIII of *P. grandis* still lacking pleopods, it implies that there are likely at least 12 zoeal stages with a duration of more than 120 days for the larval development in this species.
